# Dynamic Error Modeling and Predictive Compensation for Direct-Drive Turntables Based on CEEMDAN-TPE-LightGBM-APC Algorithm

**DOI:** 10.3390/mi16070731

**Published:** 2025-06-22

**Authors:** Manzhi Yang, Hao Ren, Shijia Liu, Bin Feng, Juan Wei, Hongyu Ge, Bin Zhang

**Affiliations:** College of Mechanical Engineering, Xi’an University of Science and Technology, No. 58 Yanta Middle Road, Xi’an 710054, China; rh2395458313@163.com (H.R.); 13669117616@163.com (S.L.); fengbinxust@xust.edu.cn (B.F.); weij@xust.edu.cn (J.W.); ghy-xkd@xust.edu.cn (H.G.); zhangb@xust.edu.cn (B.Z.)

**Keywords:** direct-drive turntable, positioning error, LightGBM, predictive compensation, adaptive correction

## Abstract

The direct-drive turntable serves as the core actuator in high-precision macro-micro drive systems, where its positioning accuracy fundamentally determines overall system performance. Accurate error prediction and compensation technology represent a critical prerequisite for achieving continuous error compensation and predictive control in direct-drive turntables, making research on positioning error modeling, prediction, and compensation of vital importance. This study presents a dynamic continuous error compensation model for direct-drive turntables, based on an analysis of positioning error mechanisms and the implementation of a “decomposition-modeling-integration-correction” strategy, which features high flexibility, adaptability, and online prediction-correction capabilities. Our methodology comprises four key stages: Complete Ensemble Empirical Mode Decomposition with Adaptive Noise (CEEMDAN)-based decomposition of historical error data, development of component-specific prediction models using Tree-structured Parzen Estimator (TPE)-optimized Light Gradient Boosting Machine (LightGBM) algorithms for each Intrinsic Mode Function (IMF), integration of component predictions to generate initial values, and application of the Adaptive Prediction Correction (APC) module to produce final predictions. Validation results demonstrate substantial performance improvements, with compensated positioning error ranges reduced from [−31.83″, 41.59″] to [−15.09″, 12.07″] (test set) and from [−22.50″, 9.15″] to [−8.15″, 8.56″] (extrapolation test set), corresponding to standard deviation reductions of 71.2% and 61.6%, respectively. These findings conclusively establish the method’s effectiveness in significantly enhancing accuracy while maintaining prediction stability and operational efficiency, underscoring its considerable theoretical and practical value for error compensation in precision mechanical systems.

## 1. Introduction

With the rapid advancement of advanced manufacturing technologies, including ultra-precision machining and ultra-high-speed cutting, precision machinery systems are facing increasingly stringent requirements for motion accuracy and dynamic performance on Computer Numerical Control (CNC) turntables. The traditional “motor + reduction mechanism” drive system, plagued by inherent limitations such as bulky mechanical transmission chains, wear and tear, and high maintenance costs, can no longer meet the demanding specifications of high-end manufacturing equipment. In contrast, direct-drive motors have emerged as the core driving component in ultra-precision positioning systems, owing to their distinct advantages of compact structure, high stiffness, superior precision, and rapid response. As the key actuating element in direct-drive turntables, their motion positioning accuracy fundamentally determines the overall turntable performance. However, in practical applications, the complex coupling effects of multiple interference sources—including motor torque fluctuation [1], thermal deformation [2], load disturbance [3], and sensor error [4]—make it challenging to fully suppress turntable positioning errors through conventional closed-loop control methods. These challenges are particularly pronounced under high-dynamic working conditions, where the time-varying and non-stationary characteristics of errors become more significant, directly impacting machining quality and equipment reliability. Consequently, systematic investigation into turntable error formation mechanisms and the development of effective compensation strategies hold substantial theoretical and engineering significance for overcoming performance limitations in ultra-precision turntables and ensuring machining quality in high-end equipment applications.

To address this technical bottleneck, current research primarily follows two technical approaches: hardware compensation and software compensation. Hardware compensation focuses on accuracy improvement through motor structure optimization or sensing system enhancement, employing methods such as asymmetric magnetic circuit design for torque pulsation suppression [5], rotor surface modification for specific torque pulsation elimination [6], aerostatic bearing integration for friction reduction [7], and advanced sensing solutions including multi-reading head averaging technology [8] and self-calibrating encoders [9]. While these methods can achieve certain accuracy improvements, they simultaneously impose significantly higher requirements on mechanical hardware and substantially increase application costs. In comparison, software compensation methods based on error mapping models have gained considerable attention due to their favorable cost-accuracy balance. Typical implementations include error compensation table construction [10,11], harmonic compensation function development [12], and polynomial fitting techniques [13]. Nevertheless, such methods predominantly depend on offline calibration, making them inherently limited in adapting to time-varying error compensation demands caused by dynamic operational changes, including sudden load variations and temperature drifts during practical applications [14].

The rapid advancement of machine learning technology has introduced a novel technical pathway for error compensation, leveraging its exceptional capability in modeling complex nonlinear systems. Early research employed neural network architectures, including Back Propagation (BP) [15], Radial Basis Function (RBF) [16], and Long Short-Term Memory (LSTM) [17,18] to achieve effective positioning error prediction and compensation through establishing nonlinear mapping relationships between error data and influencing factors. Representative studies include: literature [19] utilizing an Improved Heron Eagle Algorithm (ISBOA)-optimized BP neural network to develop a compensation model for rock drilling robotic arm end-positioning errors, achieving 84.94% improvement in positioning accuracy post-compensation; and literature [20,21] implementing neural network-based thermal error modeling that significantly enhanced system performance. Recent progress in ensemble learning has demonstrated the superior capability of gradient boosting algorithms, such as eXtreme Gradient Boosting (XGBoost) [22] and Light Gradient Boosting Machine (LightGBM) [23] in short-term prediction tasks, benefiting from their efficient tree-structured modeling and robust regularization mechanisms. Notable applications include literature [24] combining Pigeon-Inspired Optimization (PIO) with LightGBM to effectively improve permanent magnet synchronous motor dynamic response characteristics, and literature [25] proposing a robust sliding window-based LightGBM model for short-term load prediction that successfully ensures stable operation of information collection and transmission systems through anomaly detection.

However, positioning error signals in practical turntable systems frequently exhibit complex non-smooth components and significant noise interference. To enhance model prediction capability for such signals, signal decomposition techniques have been increasingly adopted to preprocess the signals by decomposing them into multiple intrinsic mode functions. This approach effectively separates noise from trend components and provides clearer signal characteristics for subsequent modeling. The successful integration of decomposition techniques with machine learning has been demonstrated across various domains, including traffic flow prediction [26], load forecasting [27], financial market analysis [28], and fault diagnosis [29]. Representative applications include: literature [30] developing a sea level prediction model combining decomposition methods with machine learning algorithms, with comparative studies across different time spans demonstrating superior prediction accuracy and stability over single prediction models; Literature [31] developed an Ensemble Empirical Mode Decomposition-eXtreme Gradient Boosting (EEMD-XGBoost) hybrid model for accurate suspended sediment load prediction; and literature [32] proposed a Complete Ensemble Empirical Mode Decomposition with Adaptive Noise-Fuzzy Entropy-Improved sparrow search algorithm-Light Gradient Boosting Machine (CEEMDAN-FE-ISSA-LightGBM) framework that successfully overcomes accuracy limitations induced by non-smooth signal characteristics in power load forecasting. Notably, improved algorithms like CEEMDAN have successfully mitigated common limitations of traditional decomposition methods, including endpoint effects and mode mixing, through optimized decomposition processes. For error prediction applications, this “decomposition-modeling-integration” strategy significantly reduces signal non-stationarity, enhances model learning effectiveness, and provides novel insights for multi-source error coupling modeling.

**Figure 1:**
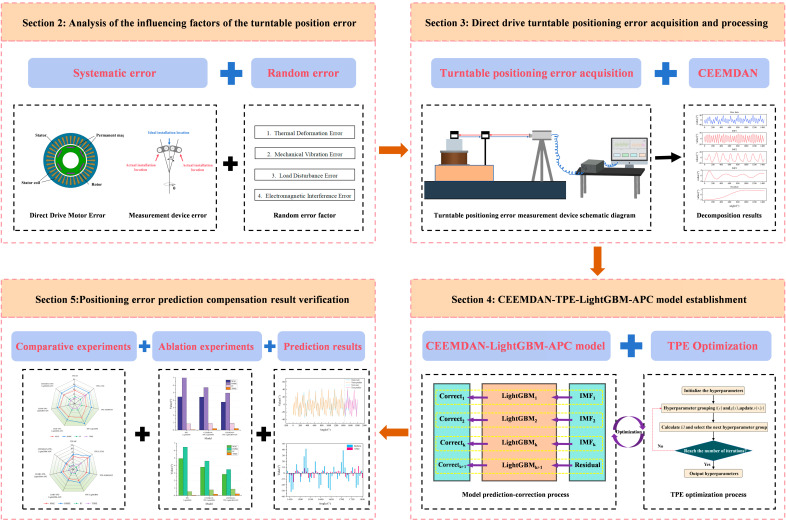
Prediction and compensation flow of positioning error of the direct-drive turntable.

Building upon this research foundation, this study proposes a novel dynamic continuous error compensation method for direct-drive turntables featuring flexibility, adaptability, and online prediction-correction capabilities, based on a “decomposition-modeling-integration-correction” strategy. The methodology comprises four key phases: (1) multi-scale decomposition of historical error data using the CEEMDAN algorithm, (2) individual modeling and prediction of each IMF component, (3) integration of component predictions to generate initial results, (4) refinement of initial predictions through an adaptive correction model to obtain final outputs. This research not only offers an innovative technical solution for overcoming accuracy limitations in ultra-precision turntables but also establishes a theoretical framework for achieving continuous error compensation, predictive control in direct-drive systems, and enhanced positioning performance in intelligent equipment. The complete research workflow is illustrated in Figure 1.

## 2. Materials and Methods

### 2.1. Direct-Drive Turntable Positioning Error Mechanism Analysis

The error factors affecting the positioning accuracy of a direct-drive turntable may be categorized into systematic errors and random errors. Systematic errors are often characterized by cyclical changes and can be eliminated. In the turntable system, these errors primarily include cogging torque fluctuations in the direct-drive motor, electromagnetic torque fluctuations, and rotary measuring element installation errors. Random errors exhibit no discernible regularity; their fluctuation characteristics are non-repeatable across repeated measurements. These errors typically arise from uncontrolled or unrecognized factors, such as thermal deformation, inherent vibration, electromagnetic interference, and environmental variations. Unlike systematic errors, they cannot be fully eliminated through correction or compensation; instead, they can only be mitigated. Thus, the total positioning error of a direct-drive rotary table may be modeled as follows:

(1) $$E_{total}=E_{systematic}+E_{random}$$

where $E_{total}$ denotes total positioning error; $E_{systematic}$ denotes systematic error factor; $E_{random}$ denotes random error factor.

### 2.2. Direct-Drive Motor Positioning Error Factor Analysis

#### 2.2.1. Direct-Drive Motor Structure

**Figure 2:**
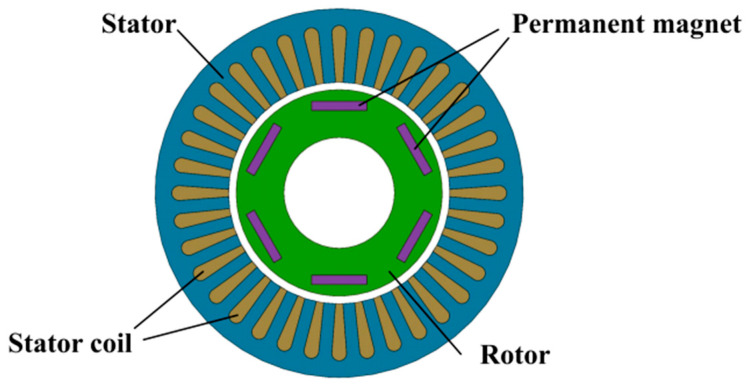
Schematic diagram of the structure of the direct-drive motor.

Direct-drive motors typically comprise a stator, a rotor, a position detection unit, a temperature control module, and a drive system. Based on rotor configuration, direct-drive motors are classified into two types: outer-rotor and inner-rotor. Outer-rotor direct-drive motors offer a large diameter, low speed, high torque, and superior heat dissipation, whereas inner-rotor motors feature a compact structure, high precision, and rapid dynamic response. This study focuses on analyzing the inner-rotor direct-drive motor, which is widely used in precision CNC machine tool turntables; its structure is illustrated in Figure 2.

#### 2.2.2. Analysis of Motor Torque Fluctuation

In direct-drive motors, torque fluctuation represents the primary factor affecting motion positioning accuracy and stability. This fluctuation primarily comprises cogging torque and electromagnetic torque, which collectively induce periodic variations in the average torque. The instantaneous output torque of the motor can be mathematically expressed as follows:

(2) $$T_{total}=T_{em}+T_{cog}$$

where $T_{total}$ is the total instantaneous torque; $T_{em}$ is the electromagnetic torque; $T_{cog}$ is the cogging torque.

Cogging torque-induced fluctuation stems from two primary sources: manufacturing imperfections in the stator and rotor, and cogging fluctuation torque resulting from rotor eccentricity. Electromagnetic torque-induced fluctuation primarily includes ripple torque resulting from the interaction between stator current harmonics and rotor magnetic field harmonics, and reluctance torque arising from asymmetrical geometric structure and magnetic circuit distribution. The unequal inductances between the d-axis and q-axis further contribute to torque fluctuation.

### 2.3. Measurement Device Installation Error Analysis

The positioning error in direct-drive turntables originates from two primary sources: motion error inherent to the direct-drive motor itself and measurement error from the measuring device. When manufacturing accuracy is ensured, measurement errors primarily occur during installation. This study employs a dual-frequency laser interferometer for turntable positioning error calibration, with particular emphasis on analyzing the principal error sources during measurement.

During laser interferometer measurements, surface irregularities (depressions or bumps) on the rotary axis mounting plane or misalignment between the table and rotation axis will induce a skew angle between the measured axis centerline and the standard indexing rotary table centerline, thereby compromising measurement accuracy.

**Figure 3:**
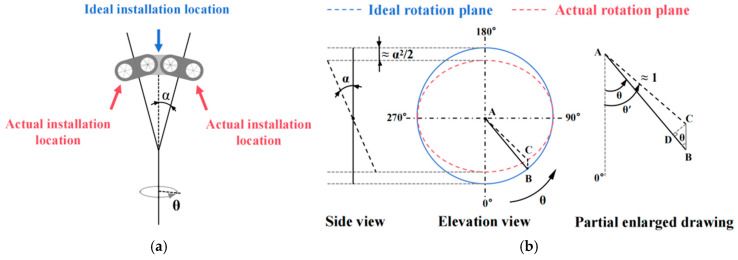
Schematic diagram of errors due to mirror tilting during the inspection process. (**a**) Misalignment of the measurement axes; (**b**) Schematic Diagram of Measurement Error Generation.

Figure 3 illustrates the motion trajectories of the standard indexing rotary table using two unit-radius curves: a solid line denoting the ideal circular trajectory (α=0) and a dashed line representing the actual elliptical trajectory (α≠0). The inclination error $E_{\theta }=\theta^{\prime }-\theta$ can be determined by first establishing a functional relationship between the elliptical and circular trajectories, followed by analytical derivation.

(3) $$E_{\theta }=\theta^{\prime }-\theta \approx CB\cdot \sin\theta =\cos\theta \cdot \alpha^{2}/2\cdot \sin\theta =\sin2\theta \cdot \alpha^{2}/4$$

where $E_{\theta }$ is the angular measurement error, and θ is the rotation angle.

### 2.4. Random Error Factor Analysis

Within ultra-precision motion systems, as performance requirements continue to advance, random errors have emerged as the primary limitation on further enhancements to motion accuracy and stability. Consequently, identifying and mitigating dominant random error sources in rotary table systems becomes essential. Primary random error sources in rotary table systems include: (1) Thermally-induced deformation: Motor-generated heat causes structural and material deformation; (2) Vibration-induced errors: System resonance creates random operational fluctuations; (3) Load disturbances: Variations in load inertia, friction torque, and related parameters induce motion deviations; (4) Electromagnetic interference: Magnetic field interactions produce motor torque fluctuations.

While random errors are inherently irreducible, their impact can be minimized through strategic approaches including: enhanced component machining and assembly precision, optimized structural design, and real-time monitoring with adaptive compensation.

### 2.5. Algorithm Principle and Model Construction

#### 2.5.1. CEEMDAN Decomposition and Reconstruction

The non-stationary nature of turntable positioning error data necessitates decomposition and reconstruction, a process that effectively reduces non-stationarity while preserving local signal features. Among conventional signal decomposition algorithms—including Empirical Mode Decomposition (EMD), Ensemble Empirical Mode Decomposition (EEMD), and Complete Ensemble Empirical Mode Decomposition (CEEMD), EMD is prone to mode aliasing; their enhanced versions (EEMD and CEEMD) mitigate this issue through Gaussian white noise addition, but inevitably introduce noise residuals. The CEEMDAN algorithm overcomes these limitations by adaptively introducing Gaussian white noise during decomposition and subsequently removing noise interference via IMF component averaging. This approach not only suppresses mode aliasing more effectively but also enhances decomposition accuracy, particularly for nonlinear and non-smooth signals. Following CEEMDAN decomposition, the signal may be represented as:

(4) $$X\left(t\right)=\sum_{k=1}^{m}\bar{IMF_{k}}\left(t\right)+r_{m}\left(t\right)$$

where $\bar{IMF_{k}}\left(t\right)$ is the mean of all $IMF_{k}$ functions at the moment, $r_{m}\left(t\right)$ is the final residual, and *m* is the number of IMFs.

#### 2.5.2. LightGBM Algorithm

Light Gradient Boosting Machine (LightGBM), developed by Microsoft in 2017, represents an enhanced version of the Gradient Boosting Decision Tree (GBDT) algorithm with superior computational efficiency. The algorithm’s key innovations include: (1) a histogram-based approach for accelerated feature splitting; (2) a leaf-wise growth strategy that replaces conventional level-wise methods, enabling more discriminative tree structures at equivalent depths; (3) integration of Exclusive Feature Bundling (EFB) and Gradient-based One-Side Sampling (GOSS), collectively enhancing training efficiency. These advancements enable LightGBM to achieve substantial reductions in both memory usage and computational time without compromising prediction accuracy. Relative to XGBoost, the leaf-wise strategy yields higher accuracy with fewer iterations; compared to Random Forest (RF), it demonstrates greater speed advantages for large-scale datasets.

#### 2.5.3. TPE Algorithm

Given that LightGBM’s performance is critically dependent on hyperparameter selection, while traditional optimization methods present notable limitations: grid search’s computational complexity scales exponentially with dimensionality; random search fails to leverage historical information; and genetic algorithms exhibit slow convergence rates. By contrast, Bayesian optimization offers superior efficiency through Gaussian process surrogate models. This study employs the Tree-structured Parzen Estimator (TPE) algorithm, a Bayesian optimization variant, to optimize LightGBM’s key hyperparameters in computationally demanding scenarios, achieving comparable optimization quality with substantially fewer evaluations.

The root mean square error (RMSE) of the validation set serves as TPE’s objective function for iterative optimization.

(5) $$f\left(x\right)=\frac{1}{m}\sum_{i=1}^{m}\sqrt{\frac{1}{M_{i}}\sum_{j=1}^{M_{i}}{\left(y_{i,j}-{\hat{y}}_{i,j}\right)}^{2}}$$

where m is the number of IMFs, $M_{i}$ is the number of samples in the *i*th IMF validation set, $y_{i,j}$ and ${\hat{y}}_{i,j}$ are the true and predicted values, respectively.

#### 2.5.4. Adaptive Prediction Correction Strategy

During turntable positioning error prediction, the error variation exhibits non-stationary characteristics due to mechanical wear, environmental disturbances, and other uncertainty factors. Conventional prediction methods typically depend on fixed parameters, resulting in prediction distortion and error accumulation. To overcome these limitations, this study introduces an adaptive prediction correction strategy combining percentile and exponential smoothing methods. The proposed approach dynamically detects anomalies and performs online correction of prediction results, thereby enhancing model accuracy and robustness against multiple error factors in practical applications. This strategy provides an effective solution for complex data environments while ensuring prediction reliability. The implementation procedure consists of the following steps:

(1) Define dynamic scaler

The non-smooth characteristics of turntable errors render fixed-threshold anomaly detection susceptible to misjudgment: an excessively high threshold fails to identify genuine anomalies, whereas an overly low threshold triggers frequent false alarms. Consequently, a dynamic threshold detection method is required to adaptively adjust detection sensitivity based on data variations, thereby preventing performance degradation associated with rigid preset parameters.

First, compute the standard deviation σ of the error series $\left\{e_{t}\right\}$ within a sliding window of size n:

(6) $$\sigma^{\left(t\right)}=\sqrt{\frac{1}{n-1}\sum_{i=1}^{n}{\left(e_{i}-\bar{e}\right)}^{2}}$$

where $\bar{e}$ is the mean value of the error.

To maintain an adaptive balance between error fluctuation and detection sensitivity, the system dynamically adjusts its judgment criteria based on the monitored error fluctuation (σ). When σ increases indicating drastic fluctuations, the scale parameter is decreased to relax threshold sensitivity and accommodate larger deviations; conversely, for decreasing σ values, sensitivity is enhanced to precisely detect minor anomalies. The scale parameter is constrained to the interval $\left[\min\_scale,\max\_scale\right]$ using a clip function. The parameter adaptation follows these rules:

(7) $$scale^{\left(t+1\right)}=clip\left(\frac{2}{\sigma^{\left(t\right)}+\varepsilon },\min\_scale,\max\_scale\right)$$

where: $\varepsilon =1\times 10^{-8}$ is a very small constant that avoids a zero denominator.

(2) Predictive outlier detection

Initially, using historical window data, compute the mean (μ) and standard deviation (σ). Subsequently, conduct anomaly detection to assess whether the predicted value lies outside the normal range. If this condition holds, the predicted value is identified as anomalous and requires correction.

(8) $$If\left|p_{t}-Q_{h}\left(q\right)\right|>scale\cdot \sigma \Rightarrow Anomaly$$

where $p_{t}$ is the current predicted value for that position, $Q_{h}\left(q\right)$ represents the lower quartile of the historical data (default q=0.25), scale is the scaling factor, and the initial scaling factor $scale^{\left(0\right)}=2$.

(3) Corrective treatment of outliers

Following anomaly detection, the identified outliers undergo smoothing correction to better align with the historical trend while minimizing anomaly-induced distortions. The proposed model employs Exponentially Weighted Moving Average (EWMA) for smoothing correction of anomalous predictions. Given the most recent historical value $h_{t-1}$ and smoothing factor α, the EWMA-adjusted prediction value $s_{t}^{\left(k\right)}$ is computed as:

(9) $$s_{t}^{\left(k\right)}=\alpha \cdot h_{t-1}+\left(1-\alpha \right)\cdot s_{t}^{\left(k-1\right)}$$

where k is the number of smoothing iterations (set to 3).

Following each adjustment, the error improvement $\Delta e=\left|p_{t}-h_{t-1}\right|-\left|s_{t}-h_{t-1}\right|$ is computed, the error sequence gets updated, and the scale parameter undergoes continuous optimization.

This adaptive prediction strategy enhances model performance for non-stationary time series by synergistically combining dynamic thresholding, anomaly smoothing, and closed-loop feedback. It addresses three key limitations of static correction methods: (1) data non-stationarity, (2) abrupt noise interference, and (3) error accumulation, thereby offering an innovative solution for reliable prediction in complex operating conditions. The core innovation manifests in:

(1) Inverse proportional design of the scaling factor: scale∝1/σ, which achieves an adaptive balance between error fluctuation and sensitivity.
(2) EWMA-based smoothing correction: Effectively mitigates short-term anomaly effects while preserving long-term trends, thus preventing trajectory deviation caused by isolated anomalies.
(3) Closed-loop feedback mechanism: Continuous optimization of parameters through error updating, forming a closed-loop self-adjusting prediction system of “prediction-correction-feedback”, so that the model continuously adapts to time-varying factors such as wear and tear of the equipment and temperature drift of the environment in long-term operation.

#### 2.5.5. CEEMDAN-TPE-LightGBM-APC Fusion Modeling

The proposed CEEMDAN-TPE-LightGBM-APC model synergistically integrates four key modules: CEEMDAN for high-performance time series decomposition, LightGBM for efficient and accurate regression prediction, TPE for hyperparameter optimization, and APC for real-time results correction. This combination enables dynamic, high-precision online prediction of non-smooth turntable positioning errors. The model operates in four stages: signal decomposition, component prediction, result integration, and adaptive correction.

In stage 1, the CEEMDAN algorithm is used to decompose the turntable positioning error sequence in multiple scales and generate k intrinsic modal function (IMF) components, which effectively separates the high-frequency and low-frequency features of the original signal.

In stage 2, for each IMF component, the LightGBM model with optimized hyperparameters based on the TPE algorithm is used for independent modeling, giving full play to the advantages of the algorithm in both efficient computation and accurate prediction in regression analysis.

In stage 3, the prediction results of each IMF component are integrated and fused to form the initial error prediction value.

**Figure 4:**
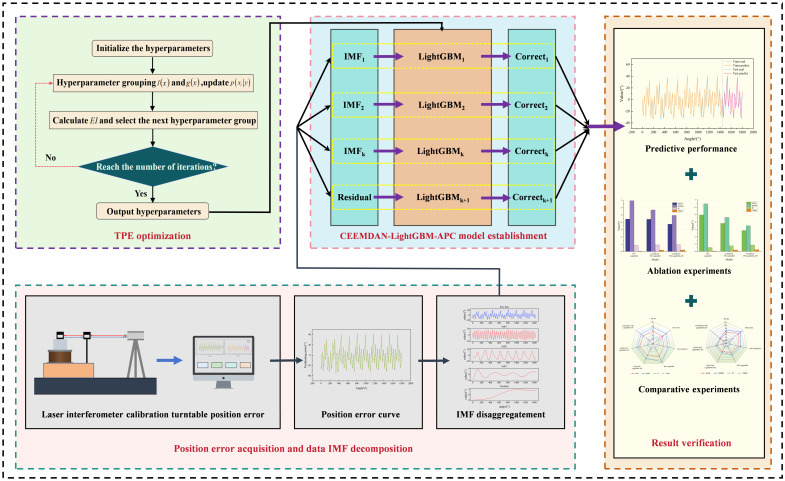
Turntable positioning error prediction process.

In stage 4, the adaptive prediction correction module is introduced to continuously detect and identify abnormal prediction points, and the smoothing correction algorithm is used to ensure that the long-term trend of the prediction results is consistent with the real process. The overall prediction process is shown in Figure 4.

As illustrated in Figure 4, the proposed framework implements a comprehensive “decomposition-prediction-integration-correction” strategy, with three principal innovations: (1) The influence of non-stationary data on the prediction model is weakened by multi-scale decomposition, which improves the adaptability of the model, (2) The independent modeling of each IMF component is able to capture both the global trend and local detailed characteristics of the data, (3) The adaptive correction mechanism enhances the stability and robustness of the prediction results by dynamically suppressing the anomalous perturbations caused by sudden changes in working conditions, while preserving the long-term trend of the data. This fusion architecture provides an effective solution for high-precision prediction of non-stationary time series data.

### 2.6. Positioning Error Experiment and Data Description

#### 2.6.1. Positioning Error Measurement and Data Description

**Figure 5:**
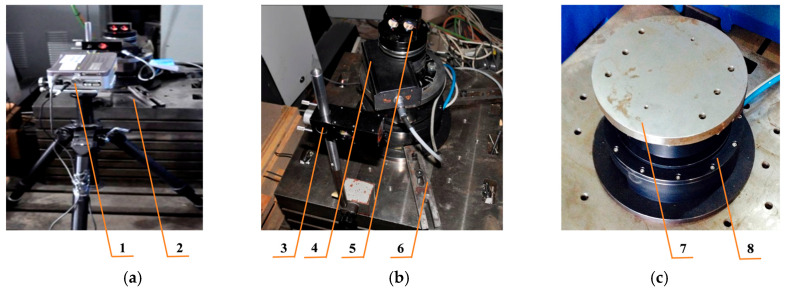
Diagram of the experimental setup for positioning error measurement. (**a**) Complete Experimental Apparatus Diagram: 1, laser interferometer; 2, experimental box. (**b**) Partial View of Turntable Measurement: 3, interference mirror; 4, calibration axis; 5, reflex mirror; 6, Fixtures. (**c**) Direct-Drive Turntable Structure Diagram: 7, rotation workbench; 8, Direct Drive Motor.

**Figure 6:**
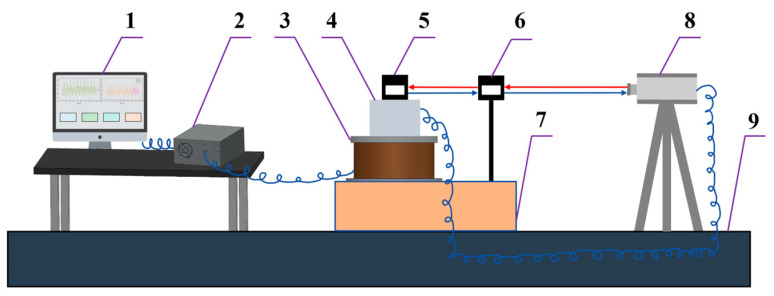
Schematic diagram of the experimental setup for positioning error measurement: 1, computer; 2, machine controller; 3, turntable; 4, calibration axis; 5, reflex mirror; 6, interference mirror; 7, experimental box; 8, laser (Renishaw’s dual-frequency laser interferometer ML10); 9, vibration isolation.

To verify the effectiveness of the proposed error prediction and compensation method, an experimental platform (Figure 5) was constructed. To minimize the impact of uncertain factors such as vibration, noise, and temperature change on the test, the turntable was mounted on a vibration isolation platform within a temperature-controlled laboratory, and a laser interferometer with a calibrated rotary device was employed to detect and calibrate for the turntable’s positioning error. The primary experimental equipment included a DM1C-004 direct-drive motor, an ML10 dual-frequency laser interferometer, and a Clipper multi-axis controller. A schematic diagram of the experimental system is provided in Figure 6.

**Figure 7:**
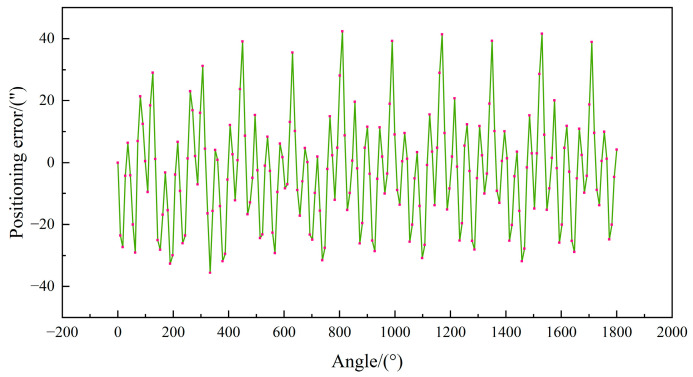
Positioning error raw measurement data.

Following calibration of the experimental platform, discrete sampling of bidirectional positional errors was performed on the turntable. To mitigate measurement uncertainties, particularly environmental temperature and humidity variations affecting system precision, the laser interferometer’s integrated temperature and humidity compensation modules were mounted on the side panel of the experimental chamber. Additional countermeasures included: (1) minimizing the distance between the laser emitter and reflector to reduce optical interference, and (2) isolating the system on a vibration-damping platform to eliminate mechanical disturbances. The sampling protocol covered a 0–1800° range with 9° intervals, acquiring bidirectional positioning error data at each measurement point. To further suppress uncertainty effects, the arithmetic mean of forward and reverse angular position errors was adopted as the raw dataset (n = 201 samples). As illustrated in Figure 7, the uncompensated positioning errors reached a maximum magnitude of 42.34″, demonstrating the necessity for error compensation.

#### 2.6.2. Dataset Construction and Partitioning

This study employs the following experimental design to systematically evaluate model performance: First, global preprocessing was applied to the full-range data (0–2160°), incorporating outlier removal via the 3σ criterion and normalization using the training set’s extremal values. Subsequently, the sliding window method was utilized to construct the dataset, where data sampled at 9° intervals from 0° to 1440° (the first 80%) comprised the training set, while data from 1440° to 1800° (the remaining 20%) formed the test set. To assess generalization capability, additional full-cycle data (1800–2160°) sampled at 10° intervals were processed with identical preprocessing parameters for extrapolation testing. This design facilitates a comprehensive evaluation of the model’s learning capacity within known data ranges and its predictive robustness across varying sampling intervals and unobserved data segments.

#### 2.6.3. Indicators for Model Evaluation

To comprehensively evaluate the error model’s prediction and compensation performance, this study adopts multi-dimensional evaluation indexes: (1) Mean Absolute Error (MAE) to reflect the overall bias level; (2) Root Mean Square Error (RMSE) to characterize the prediction stability, which is sensitive to the outliers; (3) Coefficient of Determination (R²) to quantify the model’s explanatory power; and (4) Runtime (TIME) to assess the computational efficiency. The index system realizes a comprehensive performance assessment in four dimensions: accuracy, robustness, explanatory power, and efficiency, defined as follows:

(10) $$MAE=\frac{1}{n}\sum_{i=1}^{n}\left|y_{i}-{\hat{y}}_{i}\right|$$

(11) $$RMSE=\sqrt{\frac{1}{n}\sum_{i=1}^{n}{\left(y_{i}-{\hat{y}}_{i}\right)}^{2}}$$

(12) $$R^{2}=\sum_{i=1}^{n}{\left({\hat{y}}_{i}-\bar{y}\right)}^{2}/\sum_{i=1}^{n}{\left(y_{i}-\bar{y}\right)}^{2}$$

(13) $$TIME=times_{end}-times_{stare}$$

where n denotes the number of error samples, $y_{i}$ denotes the true value of position error, ${\hat{y}}_{i}$ denotes the predicted value of position error, and $\bar{y}$ denotes the true mean value of position error.

## 3. Results

To validate the proposed turntable error prediction method, we conducted experimental studies using historical positioning error measurements. Initially, the raw error signal was adaptively decomposed into multiple intrinsic mode functions (IMFs) and residual components via the CEEMDAN method. Subsequently, training datasets for each IMF component were constructed using the sliding window method. Parameter sensitivity analysis determined an optimal window length of 10 with an increment of 1, where 10 consecutive measurements serve as inputs for predicting the subsequent measurement. For each IMF component, we implemented TPE-optimized LightGBM models. Hyperparameters were adaptively selected using the last 30% of training data as validation, with RMSE minimization as the objective. Additionally, multithreaded parallel computing was employed to enhance training efficiency. The final prediction integrated all component results, followed by adaptive correction to identify and smooth anomalies. This process mitigates single-point anomaly interference while maintaining long-term prediction stability.

All experiments were performed on a Windows 10 system with the following hardware specifications: an Intel^®^ Core™ i5-10210U processor (1.60 GHz), NVIDIA GeForce MX250 graphics card, and 16.0 GB RAM.

### 3.1. CEEMDAN Decomposition Results

**Figure 8:**
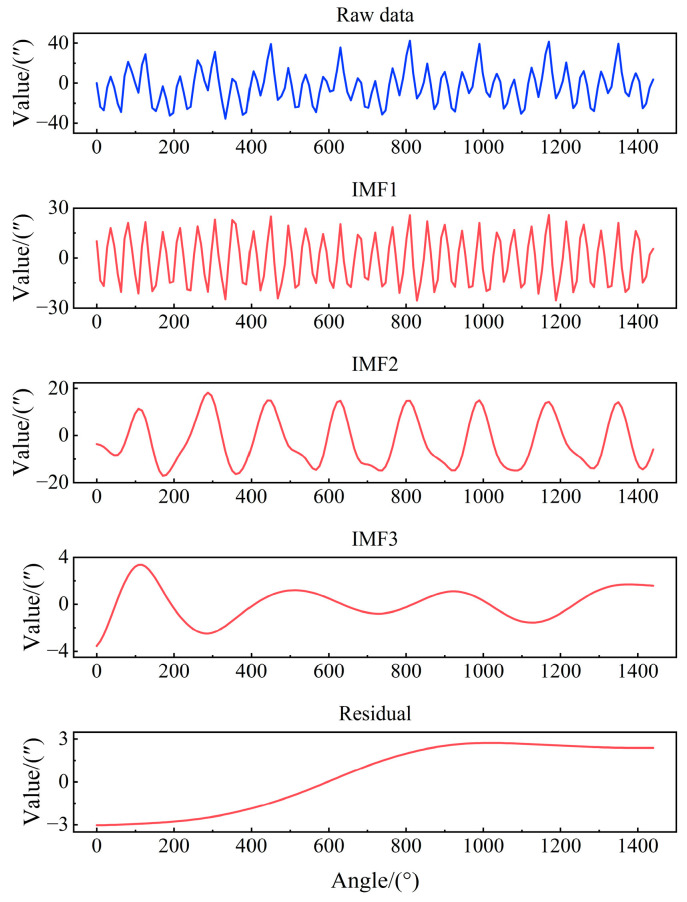
Positioning error decomposition results.

Figure 8 presents the intrinsic mode function (IMF) components and residual (Res) curves generated through CEEMDAN decomposition of the turntable positioning error sequence. The results demonstrate stable frequency characteristics across all components without modal aliasing, with low-frequency components and residuals effectively capturing the overall trend observed in the original error sequence.

### 3.2. Hyperparameter Optimization

The performance of the LightGBM model is critically dependent on its hyperparameter selection. Selecting an optimal hyperparameter combination is therefore essential for maximizing model prediction performance. We optimized the ensemble model’s key hyperparameters by minimizing the validation set RMSE over 100 iterations. The resulting optimal parameters (Table 1) were obtained while maintaining default values for other parameters.

### 3.3. Model Ablation Experiment

**Figure 9:**
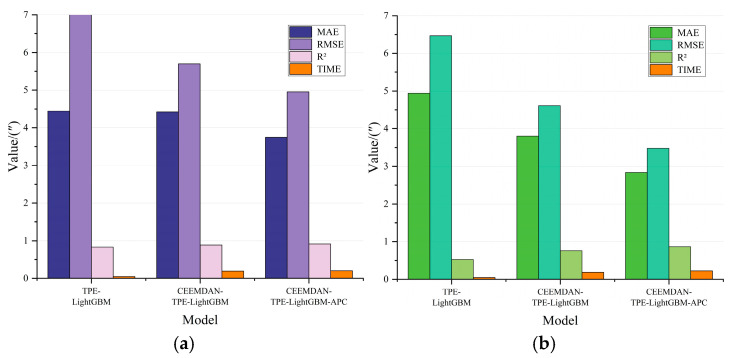
Comparison of ablation experiment model metrics. (**a**) Comparison of test set model metrics; (**b**) Comparison of extrapolation test set model metrics.

As illustrated in Figure 9, the TPE algorithm progressively enhances the overall prediction performance via hyperparameter optimization and adaptive prediction correction. With specific metrics detailed in Table 2, the CEEMDAN-TPE-LightGBM-APC model achieves optimal predictive performance, effectively balancing both accuracy and computational efficiency.

**Figure 10:**
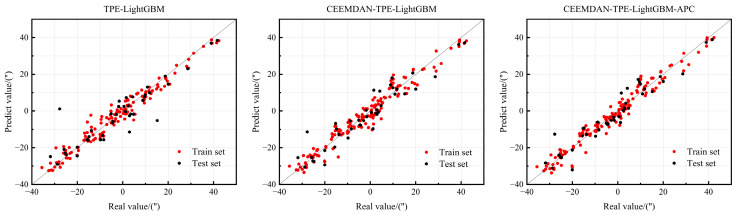
Comparison of model-predicted and actual values.

Figure 10 illustrates the correlation between predicted and actual values across the models, with red points denoting training set results and black points indicating test set predictions. Proximity to the diagonal (black dashed line) indicates superior predictive performance. As evident from the distribution, scatter points for all three ensemble learning models align closely with the diagonal. In summary, all three ensemble learning models demonstrate robust learning capacity, predictive accuracy, and generalization capability; however, the CEEMDAN-TPE-LightGBM-APC model exhibits superior performance within this study’s dataset.

### 3.4. Model Comparison Experiments

**Figure 11:**
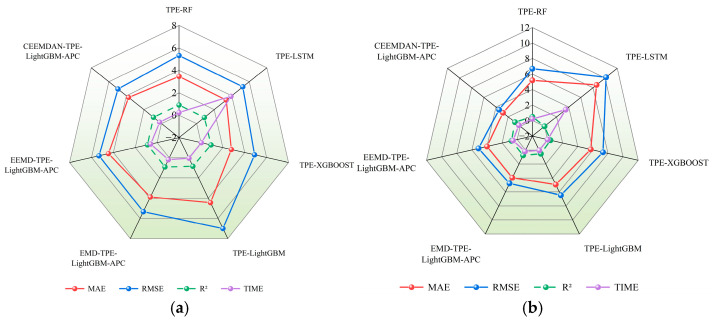
Comparison of experimental model prediction results. (**a**) Comparison of test set model metrics; (**b**) Comparison of extrapolation test set model metrics.

To further validate the predictive accuracy and stability of the CEEMDAN-TPE-LightGBM-APC model for turntable positioning errors, six benchmark algorithms (TPE-RF, TPE-LSTM, TPE-XGBOOST, TPE-LightGBM, EMD-TPE-LightGBM-APC, and EEMD-TPE-LightGBM-APC) were compared in a comprehensive study of turntable positioning error prediction. Each model’s TPE optimization targeted RMSE minimization on the validation set, with comparative results presented in Figure 11 and specific metrics detailed provided in Table 3.

The experimental results demonstrate that across all four evaluation metrics, the “decomposition-prediction-integration-correction” strategy yields superior predictive performance compared to standalone models. Specifically, CEEMDAN’s precise decomposition capability preserves data features more completely and accurately, outperforming both EMD and EEMD in predictive accuracy. For localization error prediction using individual models, LightGBM, XGBoost, and Random Forest demonstrate greater computational efficiency than LSTM while maintaining comparable accuracy. Moreover, considering both prediction stability on unseen data and practical requirements for efficient incremental learning, LightGBM surpasses both XGBoost and Random Forest in meeting online error prediction requirements.

### 3.5. Overall Model Prediction Results

**Figure 12:**
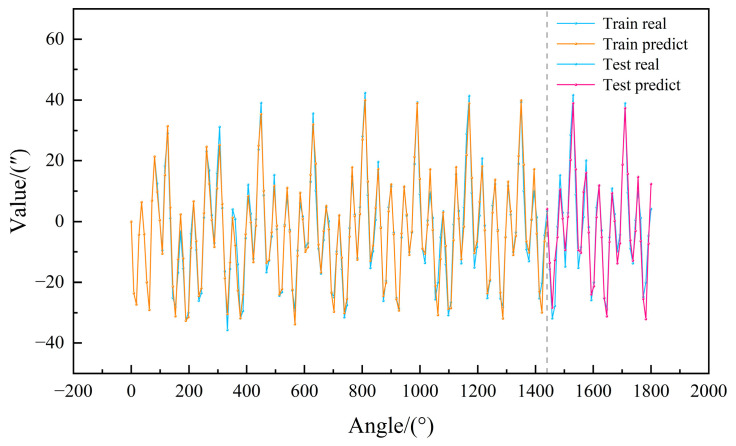
Overall prediction effects of the model.

As evidenced in Figure 12, the comprehensive predictive performance of our CEEMDAN-TPE-LightGBM-APC model across the experimental dataset reveals significant insights, where results left of the demarcation line demonstrate training set convergence while right-side results validate test set generalization. Quantitative analysis confirms the framework’s effective capture of nonlinear dynamics, with strong generalization capability (test MAE = 3.7491″), thereby demonstrating both theoretical validity and practical utility for high-precision turntable error compensation.

**Figure 13:**
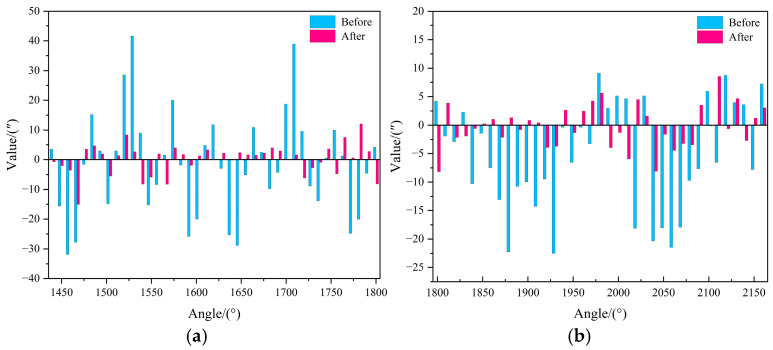
Comparison of model-predicted effects before and after compensation. (**a**) Comparison of test set model metrics; (**b**) Comparison of extrapolation test set model metrics.

To assess the enhancement in positioning accuracy achieved through error prediction compensation in the turntable system, we present a before-and-after comparative analysis of system performance in Figure 13, which shows that the error prediction compensation significantly reduces the positioning error (where ↓ indicates error reduction) and improves the smoothness of the system, with detailed metric variations provided in Table 4.

## 4. Discussion

In this study, a comprehensive experimental validation was conducted following the research design, including model ablation experiments, comparative model evaluations, and pre-/post-compensation effectiveness analyses. The key findings are summarized as follows:

(1) In the model ablation experiment, the effectiveness of TPE hyperparameter optimization, CEEMDAN signal decomposition, and APC adaptive prediction correction module is verified, respectively, and the overall prediction performance of the model is gradually improved.
(2) In the model comparison experiments, six mainstream prediction and decomposition algorithms, namely, TPE-RF, TPE-LSTM, TPE-XGBOOST, TPE-LightGBM, EMD-TPE-LightGBM-APC, and EEMD-TPE-LightGBM-APC, are used to compare with the proposed model of this study, and verify the model of this study in terms of effectiveness and superiority in the application of turntable error prediction and compensation.
(3) Comparison of the effect before and after model prediction and compensation as shown in Table 4, after the model prediction and compensation of localization error, the localization accuracy is significantly improved, and the range of localization error on the test set (9° interval) is reduced from [−31.83″, 41.59″] to [−15.09″, 12.07″], and the standard deviation is reduced from 17.16″ to 4.95″, which is a decrease of 71.2%; Meanwhile, the range of turntable positioning error on the extrapolated test set (10° interval) is reduced from [−22.50″, 9.15″] to [−8.15″, 8.56″], and the standard deviation is reduced from 9.44″ to 3.68″, with a reduction of 61.0%, which proves the validity and reasonableness of the predictive compensation of turntable positioning error.

The experimental results demonstrate that our proposed dynamic continuous error compensation model based on the “decomposition-prediction-integration-correction” strategy exhibits remarkable effectiveness and advantages in predictive compensation and motion control for direct-drive rotary tables. The key advantages are summarized as follows: (1) It achieves superior prediction accuracy and performance while maintaining an optimal balance between prediction stability and operational efficiency; (2) Compared with conventional hardware compensation and fixed-algorithm compensation methods, it demonstrates enhanced application advantages and greater flexibility; (3) As an online active compensation approach rooted in mechanistic analysis of positioning errors in direct-drive rotary tables, it proactively compensates for errors rather than passively tracking them post-occurrence, thereby offering better timeliness and practical effectiveness; (4) The adaptive prediction correction mechanism coupled with model parameter updating effectively suppresses the impact of abnormal disturbances caused by sudden working condition changes on prediction performance. This enables the system to adapt to time-varying errors and maintain long-term stable operation under actual working conditions.

(4) Compared with other studies, this study achieved a higher degree of error compensation, as shown in Table 5. This error compensation method has a better error compensation effect.

These experimental results demonstrate that the proposed methodology establishes a robust technical foundation for high-precision error prediction, compensation, and motion control in precision mechanical systems. Particularly for applications requiring micrometer-level accuracy and operational reliability, this approach offers both significant theoretical implications and practical value.

## 5. Conclusions

As the core execution part in high-precision positioning systems, direct-drive turntables critically determine overall system performance through their positioning accuracy. Addressing the limitations of traditional error compensation methods in adaptability and generalization capability, this study proposes an innovative CEEMDAN-TPE-LightGBM-APC prediction model based on a “decomposition-prediction-integration-correction” framework. The model achieves: (1) multi-scale feature extraction via CEEMDAN, (2) efficient modeling through TPE-optimized LightGBM, and (3) adaptive prediction refinement using the APC module, collectively enhancing prediction accuracy while maintaining computational efficiency. Experimental validation on test and extrapolation datasets demonstrates significant improvements: positioning error ranges narrow from [−31.83″, 41.59″] to [−15.09″, 12.07″] (test set) and from [−22.50″, 9.15″] to [−8.15″, 8.56″] (extrapolation test set), with standard deviation reductions of 71.2% and 61.0%, respectively. These results confirm the model’s dual capability in extreme error suppression and accuracy enhancement while preserving prediction stability.

Comparative analysis with mainstream machine learning models reveals the proposed method’s comprehensive advantages in accuracy, stability, and operational efficiency. The solution demonstrates both precise historical data fitting and rapid adaptation to real-time variations through its dynamic update mechanism, achieving optimal accuracy-timeliness balance for short-term prediction scenarios. This provides an effective approach for online turntable error compensation and engineering implementation.

Nevertheless, the current model architecture requires further simplification and optimization to achieve efficient deployment. In future work, we plan to develop hardware solutions, such as an FPGA, to enhance its online operational efficiency. Additionally, our ongoing research will integrate a more comprehensive set of influencing factors to further improve the model’s overall performance.

## Figures and Tables

**Table 1:** Hyperparameter optimization results.

Parameter Name	Search Scope	Final Parameter
learning_rate	0.01~0.2	0.189
max_depth	1~5	4
num_leaves	20~100	83
feature_fraction	0.5~1	0.772
bagging_fraction	0.5~1	0.849
bagging_freq	1~5	2
reg_alpha	0~1	0.958
reg_lambda	0~1	0.532

**Table 2:** Results of model ablation experiments.

Model	Performance Metrics							
	Test Set (9°)				Extrapolation Test Set (10°)			
	MAE (″)	RMSE(″)	R²	TIME (s)	MAE (″)	RMSE(″)	R²	TIME (s)
TPE-LightGBM	4.4440	7.0053	0.8334	0.0477	4.9425	6.4693	0.5261	0.0469
CEEMDAN-TPE-LightGBM	4.4182	5.6978	0.8898	0.1966	3.8017	4.6113	0.7592	0.1847
CEEMDAN-TPE-LightGBM-APC	3.7491	4.9532	0.9167	0.2065	2.8386	3.4807	0.8628	0.2216

**Table 3:** Results of model comparison experiments.

Model	Performance Metrics							
	Test Set (9°)				Extrapolation Test Set (10°)			
	MAE (″)	RMSE(″)	R²	TIME (s)	MAE (″)	RMSE(″)	R²	TIME (s)
TPE-RF	3.4626	5.3175	0.9040	0.2324	5.1921	6.6712	0.4960	0.2131
TPE-LSTM	3.3701	5.2644	0.8830	3.9130	8.5916	9.1631	0.0003	3.5000
TPE-XGBOOST	2.7778	4.8977	0.9185	0.0322	5.7221	7.3458	0.3889	0.0242
TPE-LightGBM	4.4440	7.0053	0.8334	0.0477	4.9425	6.4693	0.5261	0.0469
EMD-TPE-LightGBM-APC	3.8851	5.3311	0.8990	0.1916	3.9564	4.7780	0.7523	0.1922
EEMD-TPE-LightGBM-APC	4.4521	5.3101	0.8822	0.5864	3.9962	5.1215	0.7612	0.5660
CEEMDAN-TPE-LightGBM-APC	3.7491	4.9532	0.9167	0.2065	2.8386	3.4807	0.8628	0.2216

**Table 4:** Comparison of model-predicted effects before and after compensation.

Performance Metrics		Effect Comparison		
		Before Compensation	After Compensation	Variation
Test set (9°)	Error range	[−31.83″, 41.59″]	[−15.09″, 12.07″]	—
	Standard Deviation	17.16″	4.95″	↓ 71.2%
Extrapolation test set (10°)	Error range	[−22.50″, 9.15″]	[−8.15″, 8.56″]	—
	Standard Deviation	9.44″	3.68″	↓ 61.0%

**Table 5:** This design is compared with other references on error compensation.

References	Author	Year	Error Reduction Percentage
[33]	Bucinskas V. et al.	2022	30.0%
[34]	Liu Q. et al.	2025	41.0%
[35]	Guan Y. et al.	2025	49.5%
[36]	Liu M. et al.	2025	65.6%
[37]	Liu H. et al.	2025	63.7%
This paper	Yang M. et al.	2025	71.2%

## Data Availability

The data presented in this study are available on request from the corresponding author.
